# HORMONAL THERAPY AND THE RISK OF RHEUMATIC AUTOIMMUNE DISEASES AMONG OLDER MEN WITH PROSTATE CANCER

**DOI:** 10.1093/geroni/igae098.2312

**Published:** 2024-12-31

**Authors:** Mohanad Albayyaa

**Affiliations:** The University of Texas Medical Branch, Galveston, Texas, United States

## Abstract

Prostate cancer ranks as the second most common cause of cancer death among older men. Typically diagnosed at localized stages, treatment options primarily include radiation, hormonal therapy, or surgery. Androgen Deprivation Therapy (ADT) stands as a cornerstone in prostate cancer management, despite its association with various adverse effects such as cardiovascular and autoimmune diseases. ADT alters the mechanistic and biological pathways of androgens, impacting immune cell regulation and autoimmune equilibrium by augmenting regulatory T-cell numbers, cytokines, and pro-inflammatory markers. This study aims to investigate the potential link between hormonal therapy (ADT) and rheumatic autoimmune diseases (RAD), including rheumatoid arthritis, lupus, ankylosing spondylitis, and Sjogren’s syndrome, among older men with prostate cancer. Using Texas Cancer Registry Linked to Medicare dataset, a cohort of patients aged 66 years or older, diagnosed with localized prostate cancer between 2010 and 2019, was analyzed. Patients receiving ADT were compared to those without ADT exposure, matched on a 1:1 ratio. Kaplan-Meier analysis revealed higher rates of RAD among patients receiving ADT (11%) compared to non-ADT recipients (9%). Cox proportional hazard regression confirmed a significant association between ADT use and increased RAD risk (HR =1.29, 95% CI: 1.17-1.56). This finding underscores the importance of considering the potential adverse effects of ADT, emphasizing the need for comprehensive patient counseling and careful monitoring by clinicians. The study’s results, aligned with previous research, further solidify the understanding of ADT’s adverse effects, highlighting the necessity of assessing the patterns of care and personalized treatment strategies of older patients with prostate cancer.

